# Update on 3-iodothyronamine and its neurological and metabolic actions

**DOI:** 10.3389/fphys.2014.00402

**Published:** 2014-10-16

**Authors:** Riccardo Zucchi, Alice Accorroni, Grazia Chiellini

**Affiliations:** Laboratory of Biochemistry, Department of Pathology, University of PisaPisa, Italy

**Keywords:** T1AM, 3-iodothyronamine, thyroid hormones, neuromodulation, lipid metabolism, diabetes mellitus, learning, memory

## Abstract

3-iodothyronamine (T1AM) is an endogenous amine, that has been detected in many rodent tissues, and in human blood. It has been hypothesized to derive from thyroid hormone metabolism, but this hypothesis still requires validation. T1AM is not a ligand for nuclear thyroid hormone receptors, but stimulates with nanomolar affinity trace amine-associated receptor 1 (TAAR1), a G protein-coupled membrane receptor. With a lower affinity it interacts with alpha2A adrenergic receptors. Additional targets are represented by apolipoprotein B100, mitochondrial ATP synthase, and membrane monoamine transporters, but the functional relevance of these interactions is still uncertain. Among the effects reported after administration of exogenous T1AM to experimental animals, metabolic and neurological responses deserve special attention, because they were obtained at low dosages, which increased endogenous tissue concentration by about one order of magnitude. Systemic T1AM administration favored fatty acid over glucose catabolism, increased ketogenesis and increased blood glucose. Similar responses were elicited by intracerebral infusion, which inhibited insulin secretion and stimulated glucagon secretion. However, T1AM administration increased ketogenesis and gluconeogenesis also in hepatic cell lines and in perfused liver preparations, providing evidence for a peripheral action, as well. In the central nervous system, T1AM behaved as a neuromodulator, affecting adrenergic and/or histaminergic neurons. Intracerebral T1AM administration favored learning and memory, modulated sleep and feeding, and decreased the pain threshold. In conclusion T1AM should be considered as a component of thyroid hormone signaling and might play a significant physiological and/or pathophysiological role. T1AM analogs have already been synthetized and their therapeutical potential is currently under investigation. 3-iodothyronamine (T1AM) is a biogenic amine whose structure is closely related to that of thyroid hormone (3,5,3′-triiodothyronine, or T3). The differences with T3 are the absence of the carboxylate group and the substitution of iodine with hydrogen in 5 and 3′ positions (**Figure 1**). In this paper we will review the evidence supporting the hypothesis that T1AM is a chemical messenger, namely that it is an endogenous substance able to interact with specific receptors producing significant functional effects. Special emphasis will be placed on neurological and metabolic effects, which are likely to have physiological and pathophysiological importance.

## Distribution and metabolism of T1AM

The gold standard technique for detecting T1AM is represented by mass spectrometry, coupled to an appropriate separation technique, usually HPLC. While the initial reports of endogenous T1AM in brain (Scanlan et al., 2004), heart (Chiellini et al., 2007), and blood (Braulke et al., 2008) were admittedly non-quantitative, subsequent technical improvements allowed the assay of T1AM in virtually every tissue in rodents (Saba et al., 2010), as well as in human blood (Galli et al., 2012). Tissue concentrations were found to be on the order of 1–90 pmol/g, and the highest values were detected in liver, brain, and muscle (Saba et al., 2010). The presence of endogenous T1AM at this concentration range has been subsequently confirmed in liver (Hackenmueller et al., 2012; Ghelardoni et al., 2014) and brain (Musilli et al., 2014).

In serum T1AM was measured at a concentration on the order of 0.2–0.3 nM both in rat and in human, and so it was significantly lower than tissue concentration. In another investigation T1AM was not detected in blood (Ackermans et al., 2010), but in that study a different preparation procedure was used and method sensitivity was probably too low (0.25 nM) to get positive results. On the other hand the presence of T1AM in human blood was confirmed with a chemiluminescence immunoassay (Hoefig et al., 2011), and its concentration was estimated to be much higher, namely 66 nM. While it has been hypothesized that the immunological assay may detect a fraction of T1AM which is not extracted by the preparation procedure used for mass spectrometry assays, an alternative explanation of this discrepancy is the subsequent observation that T1AM binds with high affinity to the plasma protein apoB-100 (Roy et al., 2012 see below). In fact the immunological assay was a competition assay in which T1AM labeled with horseradish peroxidase was used as a reporter and calibration curves were not obtained in whole serum (Hoefig et al., 2011). Therefore, binding of labeled T1AM to apoB-100 may have caused overestimation of endogenous T1AM.

Experiments in which cell cultures were exposed to exogenous T1AM, or isolated organs were perfused with exogenous T1AM, confirmed that T1AM is accumulated by many different cells types, including hepatocytes, cardiomyocytes, and thyrocytes (Saba et al., 2010; Agretti et al., 2011; Ghelardoni et al., 2014), although the molecular identity of the T1AM transporter(s) still remains to be clarified (Ianculescu et al., 2009; Saba et al., 2010). Consistently, radiolabeled T1AM reached virtually every organ after intravenous injection (Chiellini et al., 2012; Lee et al., 2013). Acute uptake prevailed in liver, kidney, stomach, and intestine, while after 24 h most residual T1AM was detected in liver, muscle, and adipose tissue.

The close chemical similarity with T3 induced to speculate that T1AM may be synthetized from the T3 through decarboxylation and deiodination (Scanlan et al., 2004; Ianculescu and Scanlan, 2010; Piehl et al., 2011). However, only trace amounts of T1AM were produced in cardiomyocytes exposed to T3 (Saba et al., 2010), while administration of deuterated T4 was not associated with detection of deuterated T1AM in the liver of mice treated with perchlorate and metimazole (Hackenmueller et al., 2012). In human blood, a significant correlation between T3 and T1AM was observed when these substances were assayed by mass spectrometry (Galli et al., 2012), while in thyroidectomized patients treated with synthetic T4 normal serum T1AM values were detected by chemiluminescence immunoassay, supporting the hypothesis that T1AM may be an extrathyroidal metabolite of thyroid hormone (Hoefig et al., 2011). Therefore, the biosynthetic pathway responsible for T1AM production is still uncertain.

Tissue T1AM metabolism includes oxidative deamination to 3-iodothyroacetic acid, deiodination to thyronamine, N-acetylation and esterification with sulfate or glucoronate (Pietsch et al., 2007; Piehl et al., 2008; Wood et al., 2009; Saba et al., 2010; Agretti et al., 2011; Hackenmueller and Scanlan, 2012). Either T1AM or its catabolites undergo biliary and urinary excretion, as shown by the observations performed after administration of radiolabeled T1AM (Chiellini et al., 2012; Lee et al., 2013).

## T1AM receptors and binding sites

T1AM is not a ligand for nuclear thyroid hormone receptors, but it was found to stimulate with high affinity trace-amine associated receptor 1 (TAAR1), a G protein-coupled membrane receptor (Scanlan et al., 2004). TAAR1 was identified in 2001 on the basis of its affinity for the so-called trace amines, namely β-phenylethylamine, p-tyramine, triptamine, and octopamine (Borowsky et al., 2001; Bunzow et al., 2001; Grandy, 2007). Nine different TAAR subtypes exist, and they are widely expressed in several tissues (Zucchi et al., 2006).

So far investigations on the physiological role of TAARs have focused on the central nervous system effects of TAAR1, which has been hypothesized to act as a physiological regulator of monoaminergic neurotransmission. TAAR1 is expressed in several brain areas, particularly the limbic regions and nuclei containing monoaminergic cells (Lindemann et al., 2008). In mouse, dopaminergic neuron firing in the ventral tegmental area was modulated by p-tyramine. This effect was lost in TAAR1 knockout mice (Lindemann et al., 2008) and inhibited by N-(3-ethoxy-phenyl)-4-pyrrolidin-1-yl-3-trifluoromethyl-benzamide (EPPTB), a selective TAAR1 antagonist (Bradaia et al., 2009). In addition, type 2 dopamine receptor (D2R) antagonists enhanced TAAR1-mediated increase in cAMP, possibly by disrupting TAAR1-D2R interaction (Espinoza et al., 2011). Since D2R is the main target of antipsychotic drugs such as haloperidol (Strange, 2001), the observed functional TAAR1-D2R interaction might have potential therapeutic implications for dopamine-related disorders (Revel et al., 2011). It has also been reported that TAAR1 is a target of psychotropic agents like amphetamine, methamphetamine, 3,4-methylene-dioxymetamphetamine (MDMA, known as “ecstasy”), and d-lysergic acid diethylamide (Bunzow et al., 2001), suggesting a role in neuropsychiatric disorders. Interestingly, human TAAR genes are clustered on the long arm of chromosome 6, in a region which is consistently associated with schizophrenia or bipolar affective disorder in linkage studies (reviewed by Zucchi et al., 2006).

Using cell cultures expressing heterologous TAAR1, T1AM was found to activate rat and mouse TAAR1, inducing cAMP production with EC_50_ of 14 and 112 nM, respectively (Scanlan et al., 2004). In these models T1AM was more potent than all other trace amines. Preliminary evidence that T1AM interacts with TAAR5 has been reported (Mühlhaus et al., 2013), and TAAR8 has also been suggested as a potential target, on the basis of the pharmacological effects produced in the isolated rat heart (Frascarelli et al., 2008).

TAAR1 and possibly other TAARs represent the most likely endogenous receptor(s) for T1AM. However, receptor antagonists and knockout models are available only for TAAR1, and they have not been extensively used in experimental investigations. Therefore, the role of specific TAARs in the response to T1AM remains largely speculative, and the underlying transduction pathways also require further investigations. Apart from TAARs, additional binding sites for T1AM may exist, since the comparison between tissue T1AM concentration and tissue TAAR expression revealed a clear mismatch (Chiellini et al., 2012). As a matter of fact, several candidates have been identified and are listed below. In general, the functional consequences of T1AM binding to these targets, if any, are still uncertain, and will be discussed more extensively in the subsequent sections.

Other G protein-coupled receptors might bind T1AM, although with a lower affinity than TAAR1. In particular, in COS7 cells transfected with human or mouse alpha 2A adrenergic receptor (α_2A_), Ki values in the low micromolar range were obtained (Regard et al., 2007).

The plasma protein apo-B100, a component of VLDL and LDL lipoproteins, binds T1AM with a K_D_ of 17 nM (Roy et al., 2012). This is the likely reason for the difficulty in extracting T1AM from blood samples, and for the discrepancies reported with serum T1AM assays, as discussed above. The functional implications of this interaction are uncertain, since no evidence that T1AM may modify lipoprotein function has been reported so far.

Another molecular target appears to exist in mitochondria, since T1AM modulated the activity of sub-mitochondrial particles and soluble F_1_-ATPase (Cumero et al., 2012). Functional and biochemical data suggested the existence of a high affinity binding site (affinity on the order of 50 nM), which prevents the interaction between the ATP synthase and its physiological inhibitor IF1, and a low affinity binding site (IC_50_ = 28 μ M), through which T1AM reduces enzyme activity. Consistent with these findings, T1AM has been reported to reduce oxygen consumption and increase hydrogen peroxide release in rat liver mitochondria (Venditti et al., 2011).

At concentrations in the low micromolar range, T1AM interfered with monoamine transporters, namely norpinephrine transporter, dopamine transporter and vesicular monoamine transporter 2 (Snead et al., 2007). Both competitive and non-competitive mechanisms contributed to the inhibition since T1AM increased the K_m_ and decreased the V_max_ of each transporter. Interaction with these targets might inhibit dopamine and norepinephrine reuptake as well as their transport into synaptic vesicles. It has also been reported that micromolar T1AM displaced T3 and thyroxine (T4) from their membrane transporters, namely monocarboxylate transporter 8 (MCT8), organic anion transporting polypeptide 1A2 (OATP1A2), and organic anion transporting polypeptide 1C1 (OATP1C1) (Ianculescu et al., 2009).

## Metabolic effects of T1AM

The effects that were originally reported after the administration of exogenous T1AM to rodents (50 mg/Kg i.e., 128 μmol/Kg i.p.) included transient decrease in body temperature and reduction of cardiac inotropic and chronotropic state (Scanlan et al., 2004; Chiellini et al., 2007). The former effect is not mediated by TAAR1, since it was reproduced in TAAR1 knockout mice (Panas et al., 2010), and it may be related to the inhibitory effect of mitochondrial function, which has been described above. Cardiac effects have been attributed to the modulation of ionic homeostasis, namely to a reduction of sarcoplasmic reticulum calcium release and an inhibition of potassium currents, particularly transient outward current and I_k1_ background current (Ghelardoni et al., 2009). The transduction pathway probably involves specific tyrosine kinases and/or phosphatases (Chiellini et al., 2007) and pharmacological evidence is consistent with the hypothesis that the receptor triggering this pathway belongs to the TAAR family, whose major component in rat heart appears to be TAAR8 (Frascarelli et al., 2008).

In the isolated rat heart preparation, dose-response curves were obtained, and the IC_50_ for T1AM was found to be on the order of 20–40 μ M, i.e., substantially higher than average tissue levels, which are in the range of a few picomoles per g. Thus, these actions are unlikely to be physiological, although they might be exploited pharmacologically, since exogenous T1AM protected the myocardium from ischemia-reperfusion injury (Frascarelli et al., 2011). Central nervous system protection was also reported in a stroke model, and attributed to hypothermia (Doyle et al., 2007).

On the other hand, recent investigations have described metabolic and neurological effects of T1AM, occurring at relatively low dosages. Therefore, regulation of metabolic homeostasis and of central nervous system function appear to be the best candidates in the search for the physiological effects of T1AM, and they will be reviewed more extensively.

Acute metabolic responses to systemic (intraperitoneal) administration of T1AM in Siberian hamster or mouse (50 mg/Kg i.e., 128 μmol/Kg) included a reduction in respiratory quotient from ~0.90 to ~0.70 (Braulke et al., 2008), indicating a shift in metabolic pathways from carbohydrate to lipid oxidation. Consistent with these observations, T1AM treatment caused ketonuria and a significant loss of body fat. Both in mouse and in rat, the same dose of T1AM also increased plasma glucose, and this effect was attributed to hormonal changes, since inhibition of insulin secretion and stimulation of glucagon secretion were detected (Regard et al., 2007; Klieverik et al., 2009). The use of transgenic mouse lacking α_2A_, and experiments performed in pancreatic islets with Gi protein modulators, showed a biphasic effect of T1AM on insulin secretion, namely stimulation through TAAR1 and inhibition through α_2A_, the latter prevailing under physiological conditions (Regard et al., 2007).

The results discussed above were obtained using pharmacological dosages of T1AM, namely 128 μmol/Kg *in vivo* and 10 μ M *in vitro*. However, metabolic responses, particularly increased plasma glucose and increased plasma glucagon, were also elicited by i.c.v. infusion with much lower dosages (0.5 mg/Kg i.e., 1.28 μmol/Kg), suggesting a neuroendocrine action on the hypothalamic-pituitary-adrenal axis, which is a recently identified target for several hormones, including insulin, glucocorticoids and thyroid hormone (Fliers et al., 2010). The potency of T1AM turned out to be even higher than initially thought, since in subsequent investigations plasma glucose was increased after i.c.v. injection of doses as low as 3.3 nmol/Kg (Manni et al., 2012) or 0.3 nmol/Kg (Manni et al., 2013). In the latter study brain T1AM was determined, and it was observed that effective doses increased endogenous T1AM concentration by about one order of magnitude (34-fold with 3.3 nmol/Kg T1AM).

However, the possibility of a peripheral, hormone-independent action should not be ruled out, since T1AM administration (at 0.5–1 μ M concentration) was able to increase ketone body production and to stimulate gluconeogenesis in hepatic cell lines and in perfused liver preparations (Ghelardoni et al., 2014).

Recently, Haviland et al. (2013) used a combination of analytical techniques to explore the metabolic effect of prolonged treatment with T1AM (10 mg/Kg i.e., 26 μmol/Kg i.p., for 8 days) in a spontaneously obese mouse model. Breath carbon isotope ratio (^13^CO_2_/^12^CO_2_, or δ ^13^C value) was monitored continuously by cavity ring down spectroscopy (CRDS), and plasma samples were collected and analyzed by nuclear magnetic resonance (NMR). CRDS is a non-invasive technique that can be used to assess lipid vs. carbohydrate/protein oxidation in real-time: lipids are enriched in the lighter isotope (probably because of isotopic fractionation during the pyruvate dehydrogenase reaction), so during lipolysis more ^12^CO_2_ is generated, resulting in a lower δ ^13^C value (DeNiro and Epstein, 1977; Schöller et al., 1984). Breath δ ^13^C declined shortly after T1AM injection, and NMR metabolomics confirmed the increase in lipid utilization, as revealed by elevation in plasma 3-hydroxybutyrate concentration. Increased lipolysis was independent of food consumption and it was associated with weight loss (−8.2% of initial body weight after 8 days of treatment). The effect was persistent, since 2 weeks after discontinuation of T1AM treatment mice regained only 1.8% of the lost weight. In an ongoing investigation (Chiellini et al., 2013) it has been ascertained that lipolysis and weight loss induced by 26 μmol/Kg T1AM in mice are not associated with increased plasma glucose, suggesting that lipid metabolism is a more sensitive target than carbohydrate metabolism.

These long-term effects are likely related to modulation of gene expression, since T1AM significantly modified the expression of over 350 genes in adipose tissue and over 100 genes in liver, in a direction consistent with the observed metabolic changes (Mariotti et al., 2014). Modulated genes included several members of the sirtuin family and genes playing established roles in lipolysis, beta-oxidation, adipogenesis and lipoprotein metabolism.

The molecular mechanisms underlying acute and chronic metabolic effects remain to be determined, but T1AM-triggered pathway(s) may be clinically relevant. In fact, in a small clinical series serum T1AM concentration was found to be significantly increased in type II diabetes, and T1AM levels were significantly correlated with glycated hemoglobin (Galli et al., 2012).

In general, the effects which were originally reported for T1AM, namely hypothermia and cardiac depression, were opposite to those produced by thyroid hormone, so T1AM was initially viewed as a sort of feedback effector of thyroid signaling (Liggett, 2004). However, its metabolic effects (summarized in Table 1) are to a large extent synergic with the response to thyroid hormone, which is also known to induce a lipolytic effect (Mullur et al., 2014).

**Table 1:** **Metabolic and endocrine effects of T1AM**.

• Increase in plasma glucose
• Reduced carbohydrate oxidation
• Increased gluconeogenesis
• Increased lipid oxidation
• Increased ketogenesis
• Decreased body weight in obese mice
Inhibition of insulin secretion^*^
Stimulation of glucagon secretion

*The table summarizes the metabolic effects of T1AM. The reported effects have been produced with acute or chronic administration of different concentrations of T1AM (see text for further details). The effects are due to central effects on the hypothalamic-pituitary-adrenal axis, but a peripheral component may also exist*.

* *In pancreatic islets T1AM inhibited insulin secretion through α_*2A*_ adrenoreceptor (α_*2A*_R) and stimulated insulin secretion throughtrace amine-associated receptor 1(TAAR1), the former effect prevailing under physiological conditions*.

Specific comparison between the transcriptional response to T1AM and T3 revealed that the former produced significant genomic effects, which were not reproduced by the latter (Mariotti et al., 2014). Since T1AM is possibly synthetized from T3, and T1AM may affect T3 transport and availability, it would be interesting to evaluate if some effects traditionally attributed to T3 may be directly or indirectly mediated by T1AM.

Similar considerations apply to the neurological effects of T1AM, which will be discussed in the more general context of central nervous system modulation by the thyroid hormone signaling system.

## Thyroid hormone and the central nervous system: the co-transmitter hypothesis

It is well known that thyroid hormone is necessary for normal brain development, and limited thyroid hormone availability throughout fetal and neonatal periods results in mental retardation, deafness and ataxia (Schwartz, 1983). Its regulatory role in the maintenance of adult brain function has not been completely understood yet. Although it has long been known that mental retardation is a result of hypothyroidism (Jackson, 1998), different lines of research proved that significant reduction or increase in T3 levels jeopardize both cognitive and mnemonic processes when they are assessed in a clinical setting (Dugbartey, 1998) or evaluated in animal models (Alzoubi et al., 2009; Taskin et al., 2011).

Thyroid hormone can reach brain interstitial spaces through two pathways, since it can either be transported across the brain-blood barrier (BBB) or into the cerebral-spinal fluid (CSF) by specific carriers (Cheng et al., 1994). In rodents these carriers are mainly represented by MCT8, which transports both T3 and T4, and OATP1C1, which shows a higher affinity for T4 and reverse T3 (Sugiyama et al., 2003). MCT8 is expressed in choroid plexus cells, brain capillaries, and neurons but it is not present in astrocytes. It mediates T4 and T3 passage through the BBB and into the CSF (Heuer et al., 2005). OATP1C1 is present at the abluminal side of brain micro-capillary endothelium and is localized in BBB areas where acquaporin 4, a marker of astrocytes' end-feet, is also expressed. OATP1C1 is poorly expressed in adult primate BBB, suggesting a primary role for MCT8 in humans (Ito et al., 2011). Consistently, mutations in the MCT8 gene are associated with the Allan-Herndon-Dudley syndrome, an X-linked disease characterized by severe neurological involvement and reduced CSF T4 concentration (Kakinuma et al., 2005).

Even though blood-borne T3 can reach the brain tissue, nearly 80% of intracerebral T3 is produced locally from T4. Astrocytes throughout the brain and tanycytes in the third ventricle are the main site of conversion of T4 into T3, which is catalyzed by type II iodothyronine 5-deiodinase (D2) (Guadaño-Ferraz et al., 1999). At neuronal level, T3 can either cross the plasma membrane through MCT8 or OATP1C1 to interact with nuclear receptors, or modulate neuronal functions through non-genomic mechanisms. T3 is then catabolized into 3,5-T2 by type III deiodinase 3 (D3), which is specifically expressed in neurons and is also involved in the conversion of T4 into its inactive metabolite rT3 (Alkemade et al., 2005).

It has been speculated that in the brain iodothyronines may act as co-transmitters and in particular that they may modulate the response to the noradrenergic system (Gompf et al., 2010). This hypothesis is based on different lines of evidence. First of all, iodothyronines share considerable similarities with other neurotransmitters in their effects and mechanisms of action during brain development. Central neurotransmitters usually produce appreciable effects on brain even before the full development of their specific circuitry. During this early phase they act as growth factors and mitogens, stimulating cell growth and directing their migration (Lauder, 1983). Then, when neuronal processes and synapses become widely available, they bind to their specific receptors and behave as “conventional” neurotransmitters. Likewise, iodothyronines show a differential neuronal distribution in early and late development (Crutchfield and Dratman, 1983). During the first week of rat postnatal life, iodothyronines accumulate in nuclear and cytosolic fractions and affect neuronal maturation. For instance both hyper- and hypothyroidism have been demonstrated to alter dendritic arborization of granule cells in the hypothalamus of adult Wistar rats (Martí-Carbonell et al., 2012), while T3 exerts a critical role in hippocampal cell proliferation and granule cell precursor commitment (Remaud et al., 2014). At a later stage the picture changes completely: there is a geometrical growth in iodothyronine concentration at synapses and nerve terminals, whereas the presence of iodothyronines in the nucleus drops steadily, reaching a plateau 10 days after birth.

In addition, while T3 has been located in almost all regions in adult brain, reaching concentrations on the pmol/g order (Dratman et al., 1983; Morreale de Escobar et al., 1985; Pinna et al., 2002), iodothyronines concentrate specifically in the *locus coeruleus* (LC) and in collateral centers involved in noradrenergic signaling (Dratman and Crutchfield, 1978; Dratman et al., 1982). This is consistent with the peculiar location of LC, which lies on the lateral floor of the fourth ventricle, a strategic site favoring the uptake of T3 and T4, which are translocated through the choroid plexus to enter the CSF. Furthermore, noradrenaline was demonstrated to enhance the expression of D2 gene in adult rat brain (Greer et al., 1991), providing a specific mechanism through which LC could concentrate T3 in its terminals. Immunoistochemical studies confirmed that T3 is concentrated in the regions that are integrated in the noradrenergic system, where subcellular T3 distribution is quite peculiar: while in other areas T3 is mainly identified in cell nuclei, in adrenergic neurons it is located in the cell perikaria and in cell processes (Rozanov and Dratman, 1996). The latter result is consistent with the hypothesis that T3 is transported anterogradely through axonal processes in LC neurons (Gordon et al., 1999). Treatment of rat LC terminals with N-(2-chloroethyl)-N-ethyl-2-bromobenzylamine (DSP-4), a neurotoxin which specifically damages noradrenergic neurons, produced the expected selective degeneration in noradrenergic neurons, but also the loss of T3-immunoreactive cells in LC target regions.

As a matter of fact, thyroid hormone can acutely affect neuron firing. In Cornu Ammonis area 1 (CA1) T3 increased unit firing rates and magnified neuronal firing induced by norepinephrine stimulation (Caria et al., 2009). Furthermore, T3 injection in the preoptic area of hypo- and eu-thyroid rats produced significant changes in EEG patterns. In hypothyroid animals, administration of 3 μ M of T3 reduced REM sleep, whereas higher doses (10 μ M) induced an increase in the same parameter (Moffett et al., 2013). In euthyroid rats, 1–3 μ M T3 induced an increase in REM sleep and a significant decrease in slow-wave sleep duration (Martin et al., 2013). The timing of these effects suggests the involvement of non-genomic mechanisms.

Besides catecholamines, other neurotransmitters might be affected by thyroid hormone. T3, T4, and rT3 decreased GABA uptake in synaptosomes derived from rat brain (Mason et al., 1987), while T3 inhibited GABA_A_-gated chloride currents in several experimental models, namely *Xenopus laevis* oocytes (Chapell et al., 1998), synaptosomes derived from rat hippocampal neurons (Martin et al., 2004), and rat cultured hippocampal cells (Puia and Losi, 2011). Inhibition of glutamate binding to type NMDA receptor has been observed in the presence of thyroid hormone (Oguro et al., 1989), although these effects occurred at micromolar (i.e., non-physiological) concentrations. In rodent models both hypo-and hyperthyroidism increased serotonin turnover in the brainstem (Ito et al., 1977), which might result in a robust activation of 5HT_1A_ inhibitory autoreceptor in the raphe nuclei (Bauer et al., 2002), in turn leading to reduced levels of serotonin in the frontal cortex and decreased 5HT_2A_ receptor density in this area (Kulikov et al., 1999; Kulikov and Jeanningro, 2001). In this investigations only the frontal cortex was evaluated, therefore it cannot be excluded that serotoninergic transmission may be affected by thyroid hormone levels also in other neocortical areas. Indirect evidence of an action on the cholinergic system is based on the observation that in a rodent model sub-chronic and chronic T4 administration increased cholinergic activity in the frontal cortex and hippocampus, which was associated with improved spatial memory abilities, as assessed through the watermaze test (Smith et al., 2002).

## Neurological effects of T1AM

As detailed above, an intricate interplay appears to occur between neuronal function, neurotransmitter signaling pathways and specific genomic or non-genomic actions of thyroid hormone. Since 1995, it has been hypothesized that a crucial modulatory role could be played by thyroid hormone derivatives (Gordon et al., 1995), and T1AM is now emerging as a possible modulator of monoaminergic transmission and specifically of noradrenergic, dopaminergic and histaminergic circuitries.

Electrophysiological recordings performed in LC showed that the rate of discharge of adrenergic neurons was modified by local application of T1AM (10 μ M) (Gompf et al., 2010). As discussed above, there are reasons to believe that TAAR1, now considered as a specific T1AM receptor, can interact with the adrenergic system. Apart from TAAR1, additional T1AM targets might be involved in neuromodulation. As already mentioned, in synaptosomal fractions T1AM inihibited dopamine and norepinephrine transporters (Snead et al., 2007). Since these two transporters are responsible for dopamine and norepinephrine reuptake into the presynaptic terminal, the expected downstream effects of T1AM are represented at first by accumulation of extracellular monoamines, and afterwards by depletion of neurotransmitter stores and reduction of neurotransmission. Vesicular monoamine transporter 2 (VMAT2) is also subjected to inhibition. This transporter is instrumental in the translocation of neurotransmitters (i.e., dopamine, norepinephrine, serotonin, and histamine) from cytosol to synaptic vesicles, and so T1AM could deplete neurotransmitters available for synaptic transmission, although this hypothesis still needs experimental validation.

Another potential target is represented by α_2A_(Regard et al., 2007). α_2A_ plays a peculiar role in central neurotransmission, since along with α_2D_it is expressed as an inhibiting autoreceptor on noradrenergic presynaptic terminals and as a modulating heteroreceptor in serotonergic, dopaminergic, and glutamatergic neurons (Gilsbach and Hein, 2012). Cortical activation might be another consequence of α_2A_ activation: while it is widely known that LC exerts an excitatory influence on the cerebral cortex through α_1_-receptor activation (Papay et al., 2006), α_2A_ is also expressed at the cortical level (Blake et al., 1998), mainly in inhibitory interneurons, so α_2A_ stimulation induces disinhibition of the cerebral cortex (Andrews and Lavin, 2006).

In line with these results, microinjections of T1AM in the preoptic region induced a significant reduction in non-REM sleep (at doses of 1 and 3 μ g = 2.5 and 7.5 nmoles) and an increase in low and theta frequencies in the power spectrum of EEG-defined wakefulness (at a dose of 3 μ g = 7.5 nmoles) (James et al., 2013). Notably, these effects closely mirrored the effects of thyroid hormone administration. Consistent with these observations, i.c.v. injection of T1AM in a mouse model (at doses of 1.32 and 4 μ g/Kg = 3.3–10.2 nmol/Kg) produced a significant increase in exploratory activity assessed through the hole-board test (Manni et al., 2013). As it could be expected, the same results on wakefulness and motor activity were produced by the injection of norepinephrine in the preoptic region of adult rat brain (Emlen et al., 1972).

In addition to these effects, T1AM (1.32–4 μ g/Kg = 3.3–10.2 nmol/Kg) induced pro-learning and anti-amnestic responses when administered i.c.v. (Manni et al., 2013). In the object recognition task mice showed significantly enhanced exploratory preference and curiosity for the novel object, which was retained after 24 h. The passive avoidance test confirmed that T1AM favored learning both at 1 h and at 24 h after i.c.v. injection, and it counteracted the amnestic effect of scopolamine. The response to T1AM was antagonized by the monoamine oxidase inhibitor chlorgyline, which is consistent with the hypothesis of an interaction with the noradrenergic system. In fact, it is well known that LC projections to the hippocampus are involved in both formation (Sullivan et al., 1994) and retrieval (Sara and Devauges, 1988) of memories in rat models.

A recent study by Musilli et al. (2014) has suggested that T1AM main oxidative metabolite, 3-iodothyroacetic acid (TA1), may also play a role in the stimulation of memory acquisition, possibly by activating a histaminergic system, since its effect was prevented by H1 receptor antagonists. Interestingly, histamine is known to modulate the synchronization of neuron burst in CA3, which is an area of the hippocampus playing a central role in synaptic plasticity and in the formation of memory traces (Buzsaki and Draguhn, 2004).

Other interactions between T1AM signaling and histamine circuitry have been identified. Histamine has been demonstrated to modulate pain at the cortical and subcortical level, inducing hyperalgesia at low doses through H1 receptors (Malmberg-Aiello et al., 1994; Galeotti et al., 2004). In line with these observations, i.c.v. injection of TA1 (0.4 μ g/Kg) reduced the threshold to painful stimuli in mice subjected to the hot plate test, and the effect was completely abolished when TA1 was co-administered with histamine receptor antagonists and in mice lacking histidine decarboxylase, the enzyme responsible for histamine synthesis (Musilli et al., 2014).

T1AM has also important effects on the regulation of food intake. Intracerebral T1AM injection induced significant alteration in feeding behavior in fasting mice and in mice fed *ad libitum*. In the latter group, when T1AM was administered either in the arcuate nucleus (at doses of 0.12–1.2 nmol/Kg) or in cerebral ventricles (at the dose of 1.2 nmol/kg), an orexigenic effect was induced (Dhillo et al., 2009). It was also observed that exposure of hypothalamic slices to T1AM *in vitro* induced neuropeptide Y (NPY) release, suggesting that this potent orexigenic peptide is involved in the hyperphagic effect. However, in fasting mice a biphasic response was elicited by i.c.v. T1AM administration, since low dosages (1.32 μ g/Kg = 3.3 nmol/Kg) produced an anorexic effect while higher dosages (20 μ g/Kg = 51 nmol/Kg) turned out to be orexigenic (Manni et al., 2012).

Effects of chronic treatment have also been reported. In a study that was published only in abstract form, Hettinger et al. (2010) observed that chronic systemic (i.p.) administration of T1AM (31 mg/Kg per day = 79 μmol/Kg per day for 14 days) reduced food intake in mice, while no change in food assumption was observed at a lower dosage (10 mg/kg per day = 26 μmol/Kg per day for 8 days) by Haviland et al. (2013).

The mechanism of feeding modulation by T1AM is not known, but a role for histamine cannot be excluded. In fact, histaminergic neurons form a network that is involved in the balance of neuroendocrine and feeding inputs within the hypothalamus. Histamine induces suppression of food intake when interacting with the satiety center in the ventromedial hypothalamus (Ookuma et al., 1993). On the other hand, i.c.v. injection of H3 histamine receptor antagonists induce suppression of food intake (Cohn et al., 1973). Therefore, this dual action on feeding behavior might be involved in the effects of T1AM and/or its catabolite TA1 on food intake.

Most interestingly in the few investigations in which brain tissue could be assayed to determine thyroid hormone and T1AM, it was observed that after administration of effective T1AM dosages (1.3 μ g/Kg = 3.3 nmol/Kg), average brain T1AM concentration increased by about one order of magnitude over the baseline, while brain T3 and T4 concentrations were unchanged (Manni et al., 2013). So, functional effects occurred at tissue concentrations close to the physiological range. A summary of the neurological effects which have been described after T1AM administration is reported in Table 2.

**Table 2:** **Neurological effects of T1AM**.

Electrophysiological effects on the *Locus Coeruleus:*
• Increased neuronal firing
EEG patterns (microinjection in the preoptic region):
• Reduction in nREM sleep
Behavior (i.c.v. administration):
• Increase in exploratory activity
Memory (i.c.v. administration):
• Prolearning and antiamnestic effect
Pain (i.c.v. administration):
• Decreased pain threshold to hot stimuli
Food intake (acute central administration):
• In *ad libitum* fed mice: orexigenic effect
• In fasting mice: biphasic effect, with anorexic properties at low doses and orexigenic effects at higher doses
Food intake (chronic peripheral administration, i.p.):
• Anorexic effect

*The table summarizes the neurological effects of T1AM. The reported effects have been produced with acute or chronic administration of different concentrations of T1AM (see text for further details). Abbreviations: EEG, electroencephalography; i.c.v., intracerebroventricular; i.p., intraperitoneal*.

## Exploiting T1AM signaling

**Figure 1:**
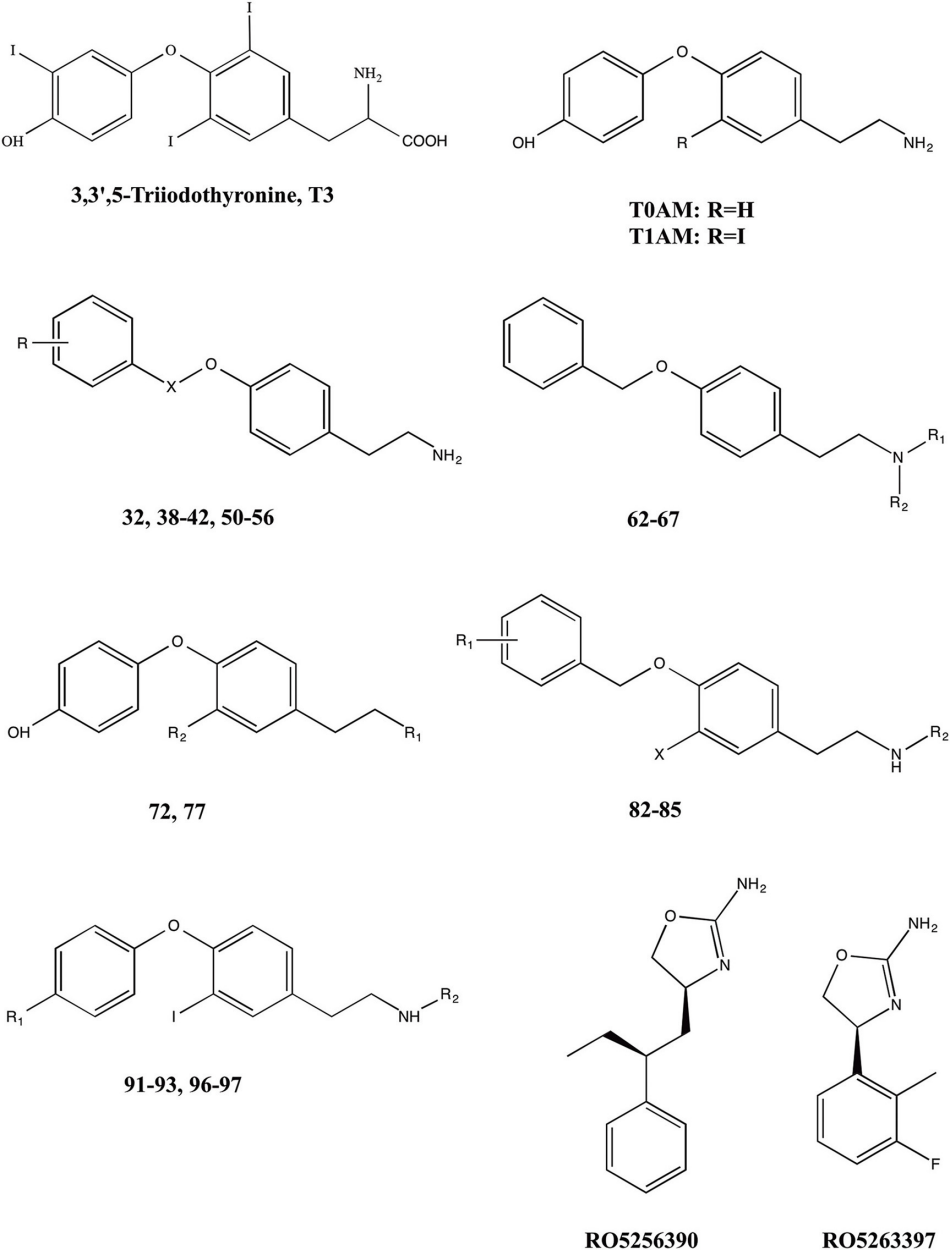
**Chemical structures of 3,5,3′-triiodothyronine (T3),endogenous thyronamines (T1AM–T0AM) and synthetic analogs**.

The different functional effects of T1AM and the widespread distribution of TAARs raise the hope that this novel signaling system may be exploited in human therapeutics. A large number of T1AM derivatives have already been synthesized and evaluated as TAAR1 agonists. As shown in Figure 1, the first generation of T1AM analogs (Hart et al., 2006) featured removal of the phenol hydroxyl, an increase in the distance between the two aryl rings, a change in the electronic and steric requirements on the aryl ring distal to the amine functionality, alkylation or modification of the amine, and replacement of the 3-iodo substituent with an alkyl group. These analogs were evaluated using the cAMP accumulation assay in cells stably expressing either rat or mouse TAAR1 (rTAAR1 and mTAAR1). Analysis of the results summarized in Table 3 suggests the following requirements for TAAR activation:

- A basic amino group at Cα is required. In the case of compound **72**, where the amine was replaced with a hydroxyl, no TAAR1 activation was observed.
- Monomethylation of the amine can be beneficial (compounds **62** and **85**) although larger alkyl groups (compounds **64-66**) and bis-alkylation (compound **67**) are deleterious.
- mTAAR1 and rTAAR1 differ with respect to their tolerance of changes in the diaryl linker, both in length and in functionality. In the case of rTAAR1 activation, derivatives **77**, **91**, and **92** were the most potent, while in the case of mTAAR1, the most effective compounds were derivatives **85**, **91**, and **92**.
- Within the thyronamine scaffold, an iodine or methyl substituent at the 3-position is optimal. Derivative **77** with a methyl group at the 3-position of the thyronamine scaffold was nearly as potent as T1AM against both rTAAR1 and mTAAR1.
- An H at the 4′-position vs OH is optimal (compound **91** was the most potent agonist of rTAAR1 and one of the most potent at mTAAR1) and substituents larger than OH are deleterious (compounds **93** and **97**).

**Table 3:**
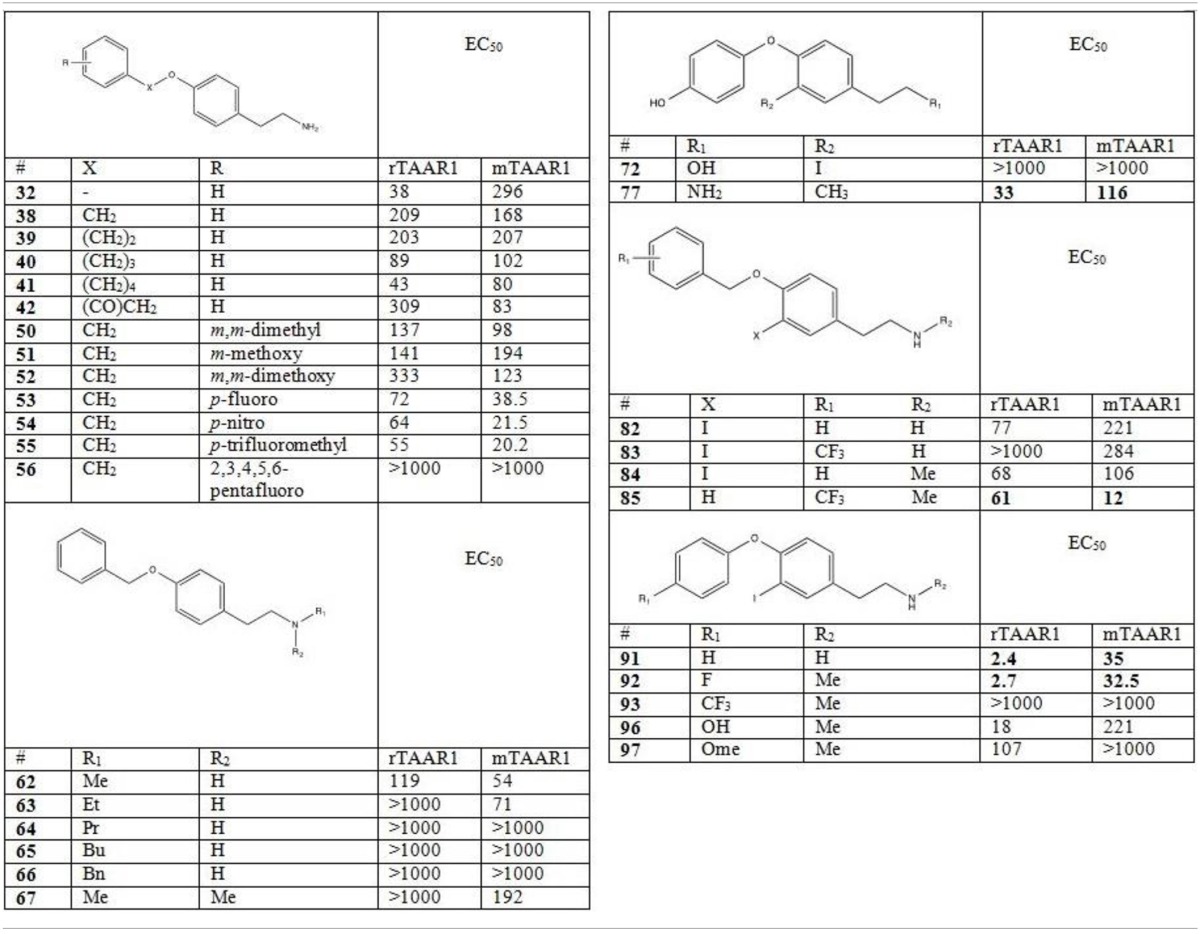
**Activity of first generation analogs of thyronamines on rTAAR1 and mTAAR1 (as reported by Hart et al., 2006)**.

*Concentrations are expressed as nM*.

The most potent derivatives reported by Hart et al. (2006), namely compounds **77**, **85**, **91**, and **92**, were also examined for hypothermia induction in mice. When administered to mice at a 50 mg/kg dose, all derivatives induced significant hypothermia within 60 min and exhibited a hypothermic induction profile analogous to T1AM. Derivative **91** was the most potent, and an ED50 of 30 μmol/kg was calculated.

On the basis of the report that TAAR1 can be activated by phenethylamine analogs like amphetamines and ergolines (Bunzow et al., 2001), an additional group of phenyltyramine derivatives was devised and analyzed by Tan et al. (2007). This investigation showed that the potency of thyronamines for both rTAAR1 and mTAAR1 can be enhanced by incorporating appropriate functionalities in the ethylamine portion of the phenoxyphenylethylamine scaffold. Even though rTAAR1 and mTAAR1 are 93% similar, the two rodent receptors have different structural preferences for this region of the scaffold, with rTAAR1 favoring unsaturated hydrocarbon groups and mTAAR1 preferring functional groups that are polar and hydrogen-bond acceptors. Analysis of single and double mutants of rat and mouse TAAR1 (Tan et al., 2009) showed that key, non-conserved specificity determinant residues in transmembranes helices 4 and 7 within the ligand binding site appear to be the primary source of the observed ligand preferences. In particular, residue 7.39 appears to dictate the specificity for a β-phenyl ring: a bulky tyrosine residue at 7.39 in mTAAR1 sterically clashed with the β-phenyl ring, whereas a smaller asparagine residue at the same location in rTAAR1 was able to accommodate a β-phenyl moiety. The lower potency of T1AM in mTAAR1 (EC_50_ = 112 nM) vs. rTAAR1 (EC_50_ = 14 nM) appeared to be caused by the presence of a tyrosine instead of a phenylalanine at residue 4.56. Despite this species variability, transforming the inner ring of the phenoxyphenethylamine scaffold into a naphthyl group, as in compound **24**, was equally beneficial to both receptors, most likely acting as an excellent isosteric replacement for the iodophenyl inner ring of T1AM (Table 4).

**Table 4:** **Activity of naphethylamine (derivative 24) on rTAAR1 and mTAAR1 (derived by Tan et al., 2007)**.

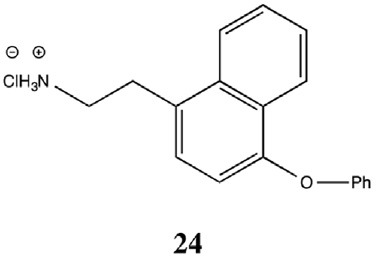			
**rTAAR1**		**mTAAR1**	
**EC_50_ ± s.e.m. (nM)**	**E_**max**_ ± s.e.m. (%)**	**EC_50_ ± s.e.m. (nM)**	**E_**max**_ ± s.e.m. (%)**
26 ± 1	113 ± 5	101 ± 22	104 ± 3

A different approach has been followed by Hoffmann-La Roche chemists, who have developed a novel series of imidazole compounds that are potent and selective partial and full agonists of the TAAR1 receptor (Galley et al., 2012; Revel et al., 2013). In rodents, activation of TAAR1 by the full agonist RO5256390 and the partial agonist RO5263397 (Figure 1), blocked psychostimulant induced hyperactivity and produced a similar activation pattern as the antipsychotic drug olanzapine. Notably, TAAR1 agonists do not induce catalepsy or weight gain; RO5263397 even reduced haloperidol-induced catalepsy and prevented olanzapine from increasing body weight and fat accumulation. In addition, TAAR1 activation promoted vigilance in rats and showed pro-cognitive and antidepressant-like properties in rodent and primate models (Revel et al., 2013).

Quite recently a preliminary report has described the synthesis of T1AM analogs in which the two aromatic rings are linked by a methylene group, the hydroxyl group (OH) at position 4′ has been replaced by an amino group (NH2), and the ethylamine side chain at position 1 has been replaced by an oxy-ethylamine side chain (Chiellini et al., 2014). Interestingly, these compounds were effective on mouse TAAR1 but in functional experiments they increased plasma glycemia and reduced cardiac output, i.e., they produced effects which are known not to be mediated by TAAR1 (Regard et al., 2007; Frascarelli et al., 2008). These observations raise the possibility that different receptors systems may be targeted by T1AM analogs and/or putative TAAR1 agonists.

## Conclusions

In conclusion, T1AM is a novel chemical messenger, that interacts with a specific G protein-coupled receptor, TAAR1, and possibly with other molecular targets. At tissue concentrations close to the physiological range it produces significant metabolic and neurological effects. From the metabolic side, it stimulates lipid catabolism and induces in general anti-insulin responses; from the neurological side, it has been reported to favor learning and memory, modulate sleep and feeding, and decrease the pain threshold. While available evidence suggests that T1AM should be regarded as a neuromodulator, the molecular details of its actions, and the underlying transduction pathways, remain to be determined.

It has been suggested, although not yet formally demonstrated, that T1AM is synthetized from T3, and some of its actions are partly synergic with, but not identical to, the known metabolic and neurological responses to thyroid hormone. So T1AM might be responsible for some effects traditionally attributed to thyroid hormone itself, and should be viewed as a component of thyroid hormone signaling. Exploring this novel aminergic system might open new perspectives in the analysis of hormonal and neuroendocrine regulation of energy balance and behavior, and provide new targets for potential therapeutic interventions in metabolic, endocrine and neurological disease.

Critical research issues for the near future include clarifying the role of TAAR1 vs other receptors in the response to T1AM and dissecting the underlying transduction pathways. In order to confirm the physiological actions of T1AM, it is also essential to reduce or abolish endogenous T1AM production by appropriate experimental interventions. The latter would require a better understanding of the biochemical pathway(s) responsible for T1AM synthesis.

### Conflict of interest statement

The authors declare that the research was conducted in the absence of any commercial or financial relationships that could be construed as a potential conflict of interest.
